# The accuracy of serum osmolarity calculation in small children

**DOI:** 10.5937/jomb0-37490

**Published:** 2023-01-20

**Authors:** Joanna Berska, Jolanta Bugajska, Krystyna Sztefko

**Affiliations:** 1 Jagiellonian University College of Medicine, Institute of Pediatrics, Clinical Biochemistry Department, Krakow, Poland

**Keywords:** calculation, children, newborns, osmolality, osmolarity, proračun, deca, novorođenčad, osmolalnost, osmolarnost

## Abstract

**Background:**

Serum osmolality can be measured (Omeas) or calculated (Ocal). Many formulas for Ocal have been already published, but data regarding the most accurate equation in small babies is not available. Thus, we aim to compare Omeas and Ocal obtained by different formulas in newborns and small children.

**Methods:**

The study included 280 serum samples taken from children, from the first day of life to 2 years (mean age 8.2 ± 7.6 months) treated in the University Children's Hospital in Krakow. The serum osmolality was measured by osmometer and calculated by 8 common formulas.

**Results:**

The mean value of Omeas (2 8 5 .8 ± 5 .1 mOsm/kgH2O) was significantly different as compared to the mean values of Ocal (p< 0.01) for all formulas, except Ocal obtained by the formula: 1.86*(N a + K) +1.15*Glu + Urea + 14. According to Bland-Altman analysis, this formula showed the best performance for estimating osmolality. In children under 3 months of life Passing-Bablok regression indicated both systematic and proportional error for results obtained by each formula compared to the measured values.

**Conclusions:**

To calculate osmolarity in children aged between 3 months and 2 years old the following equation: 1 .86*(N a + K) + 1.1 5*G lu+ U rea+ 14 might be used, whereas serum osmolality in children up to 3 month of life should be measured.

## Abstract (sr)

**Uvod:**

Osmolalnost seruma se može meriti (Omeas) ili izračunati ( Ocal). Mnoge formule za Ocal su već objavljene, ali podaci o najtačnijoj jednačini kod malih beba nisu dostupni. Stoga želimo da uporedimo O meas i Ocal dobijene različitim formulama kod novorođenčadi i male dece.

**Metode:**

Studija je obuhvatila 280 uzoraka seruma uzetih od dece, od prvog dana života do 2 godine (srednja starost 8,2 ± 7,6 meseci) lečene u Univerzitetskoj dečjoj bolnici u Krakovu. Osmolalnost seruma je izmerena osmometrom i izračunata po 8 uobičajenih formula.

**Rezultati:**

Srednja vrednost Omeas (285,8± 5,1 mOsm/ kgH2O) se značajno razlikovala u poređenju sa srednjim vrednostima Ocal (p< 0,01) za sve formule, osim Ocal dobijene po formuli: 1,86*(N a + K) + 1 ,1 5* Glu + Urea + 14. Prema Bland-Altman analizi, ova formula je pokazala najbolje performanse za procenu osmolalnosti. Kod dece mlađe od 3 meseca života Passing-Bablokova regresija je pokazala i sistematsku i proporcionalnu grešku za rezultate dobjene svakom formulom u poređenju sa izmerenim vrednostima.

**Zaključak:**

Za izračunavanje osmolarnosti kod dece uzrasta između 3 meseca i 2 godine, mogu se koristiti sledeće jednačine: 1,86*(N a + K) + 1,15*Glu + Urea + 14, dok treba meriti osmolalnost seruma kod dece do 3 meseca života.

## Introduction

Serum osmolality is important to assess waterelectrolyte balance affecting homeostasis of the body [1]. It is also helpful in the diagnosis of hyperglycaemic nonketotic coma, diabetes insipidus, syndrome of inappropriate antidiuretic hormone secretion as well as in monitoring treatment with osmotically active substances, e.g. mannitol [2]. Body water constitutes about 60% of total body weight in healthy adults, in newborns and children is even higher, ranging from 65% to 75%. Total body water contents mostly depends on body composition such as fat and muscle mass and can be estimated using bioelectrical impedance analysis or magnetic resonance [3]. Water is distributed in intracellular and extracellular spaces separated by semipermeable membranes [4]. These membranes enable the transport of substances across the phospholipid bilayer, through various mechanisms: passive diffusion (non-selective process for small molecules, which can move down their concentration gradients) and active transport (for polar substances such as ions, against the concentration gradient), which requires the activity of specific transport and channel proteins. This process uses energy stored in ATP and is highly selective, because only the appropriate size and charged ions can cross the channels. For sodium and potassium ions the specific channels have been demonstrated - the Na/K ATPase pump helps maintain a negative membrane potential and osmotic equilibrium [5]. In response to the difference in solutes concentration between the two sides of the membrane the flow of water across a plasma membrane is possible. The factor determining the volume of fluid spaces is osmolality, which is defined as a total number of osmotically active ions/particles contained in a kilogram of solvent [6]. The osmolality of a solution depends only on the number of dissociated particles, but not on their molecular weight and charge [7]. The difference in the concentrations of chemical compounds or ions on both sides of the membrane generates osmotic pressure and water movement, consistent with the concentration gradient [8]. The regulation of the composition and volume of body fluids takes place mainly with the participation of the kidneys and lungs and includes mechanisms of thirst, autoregulatory processes and humoral and nervous processes [2]. Serum osmolality is very precisely regulated by altering the amount of sodium and water excreted from the body and is maintained in a narrow range 275 to 295 mOsm/kg H_2_O [9]. Osmolality can be measured (O_meas_) using the phenomenon of changing the properties of a solvent, like freezing point, boiling point or vapour pressure, due to osmotically active substances dissolved [10] [11]. In many laboratories it is not possible to measure osmolality directly, therefore serum osmolarity is calculated (O_cal_) by different formulas, usually taking into account the concentration of osmotically active substances present in the plasma, i.e. sodium, potassium, glucose and urea [12]. Many formulas for O_cal_ have been already published [12], but data regarding the equation best for osmolarity calculation in children and clear recommendation for using any given formula in small babies is not available. Thus, the aim of the study was to compare measured osmolality and calculated osmolarity obtained by different formulas in newborns and small children.

## Material and methods

The study included 280 routine serum samples taken from children, from the first day of life to two years (140 boys and 140 girls, mean age 8.2 ± 7.6 months) treated in the University Children's Hospital in Krakow, Poland. Concentrations of serum sodium, potassium, glucose and urea were measured by dry chemistry analyser (Vitros 4600, Ortho Clinical Diagnostics Inc., Rochester, NY, USA). Using a spectrophotometric technique HIL (haemolysis, icterus and lipemia) index was measured automatically in all samples. Haemolysis leads to significant increase of potassium and according to the manufacturer (Ortho Clinical Diagnostics), samples with haemolysis index HI < 15 (equivalent to 0.15 g/L of free hemoglobin) were considered as having no haemolysis. Lipemia may affect as well as analytes determination (e.g. sodium) and osmolality measurement [13]. Lipoproteins reduce the water volume of the sample. Haemolysed and lipemic samples were excluded from the study. On the basis of clinical diagnosis of children treated in the hospital wards, it was found that the children were not poisoned with alcohol. None of the children were treated with mannitol. Within 3h of serum collection, osmolality was measured by the assessment of depression of the freezing point by osmometer model 800CLG. Each measurement was performed in duplicated and mean values were used for further analysis. Serum osmolarity was calculated by 8 common formulas provided in Table 1.

**Table 1:** Formulas used to calculate serum osmolality. Sodium (Na), potassium (K), glucose (glu) and urea are expressed in mmol/L.

No.	Formula
1	2^*^Na+Glu+Urea
2	2^*^Na+Glu
3	2^*^(Na+K)+Glu+Urea
4	1.86^*^(Na+K)+Glu+Urea+10
5	1.89^*^Na+1.38^*^K+1.08^*^Glu+1.03^*^Urea+7.45
6	1.86^*^(Na+K)+1.15^*^Glu+Urea+14
7	1.09^*^(1.86^*^Na+Glu+Urea)
8	1.86^*^Na+Glu+Urea+9

Comparison of measured (O_meas_) and calculated (O_cal_) values was performed in all samples as well as in samples taken from babies from the first day to 3 months of life (group 1, mean age 0.7 ± 0.7 months, F/M 46/49), from babies between 3 months to 1 year (group 2, mean age 6.4 ± 2.6 months, F/M 48/48) and in children between 1 and 2 years of life (group 3, mean age 18.3 ± 2.9 months, F/M 46/43). The concentrations of sodium, potassium, glucose and urea were within typical reference intervals in all children. Characteristics of the study group was presented in Table 2. The statistical analysis of obtained data was performed using software package Statistica 13 (StatSoft Inc.) and MetCalc 20.2015. The Shapiro-Wilk test was used to determine the normality of data distribution. Data are presented as median (with interquartile range). The Wilcoxon test were applied to compare the values of measured osmolality and calculated osmolarity. Correlations between O_meas_ and O_cal_ were assessed by Pearson correlation analyses. The agreement and differential bias between calculated osmolarity and measured osmolality with 95% limits of agreement was assessed by Bland-Altman method. For method comparison Passing-Bablok regression analysis was performed. The level of statistical significance was established as p less than 0.05. The total allowable error (TEa) of the mean measured value of osmolality was calculated. According to Westgard [14], TEa for serum osmolality is 1.5%. The study was approved by Jagiellonian University Bioethics Committee (Protocol No. 1072.6120.52.2021).

**Table 2:** Characteristics of the study group.

	All<br>samples<br>n = 280	Group 1<br>n = 95	Group 2<br>n = 96	Group 3<br>n = 89	p<br>within groups		
					1 and 2	2 and 3	1 and 3
Sodium<br>[mmol/L]<br>Mean ± SD	136.3 ± 2.1	136.0 ± 2.1	136.1 ± 2.1	136.8 ± 2.1	p < 0.90	p < 0.06	p <0.02
Potassium<br>[mmol/L]<br>Mean ± SD	4.91 ± 0.55	5.14 ± 0.61	4.90 ± 0.50	4.69 ± 0.43	p < 0.003	p < 0.03	p < 0.00003
Glucose<br>[mmol/L]<br>Mean ± SD	4.8 ± 0.9	4.5 ± 0.9	4.9 ± 0.9	4.9 ± 1.0	p < 0.02	p < 0.98	p < 0.03
Urea<br>[mmol/L]<br>Mean ± SD	3.4 ± 1.6	3.3 ± 1.8	3.0 ± 1.2	4.1 ± 1.5	p < 0.52	p < 0.0001	p < 0.001

## Results

**Figure 1:**
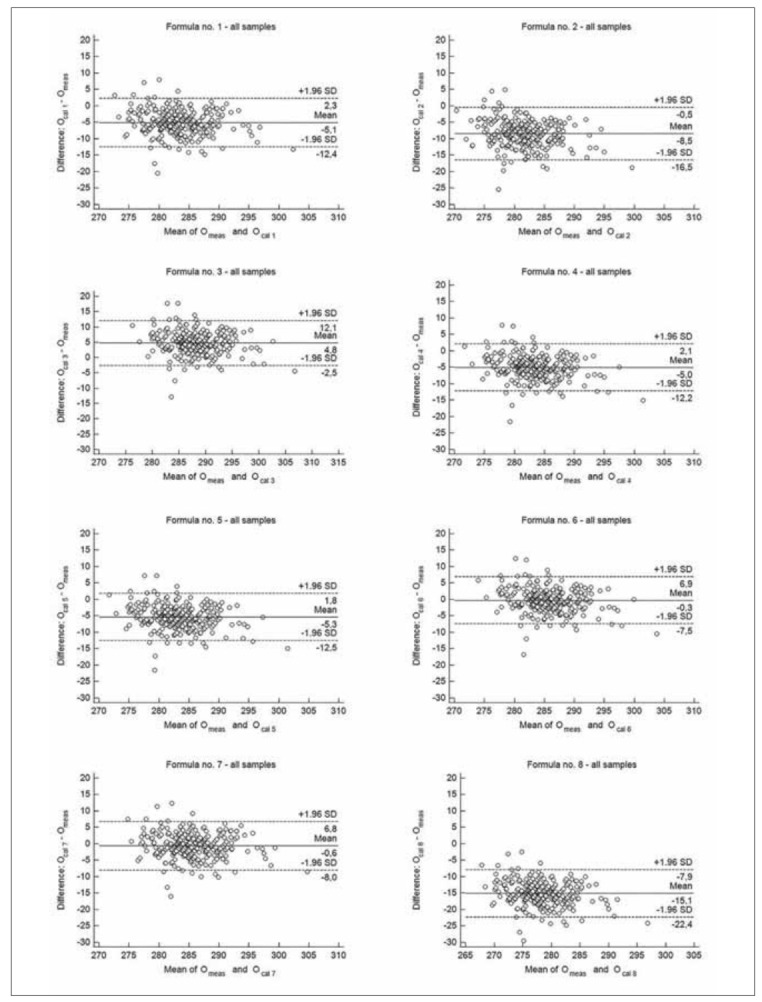
The Bland-Altman plots for the difference between measured osmolality (O_meas_) and calculated by different formulas (O_cal 1-8_) – all samples.

Measured osmolality (O_meas_) and calculated osmolarity (O_cal_) values, obtained by eight formulas, are shown in Table 3. When all samples were analysed, the median value of O_meas_ (286.0 mOsm/kg H_2_O) was significantly different as compared to the median values of O_cal_ (p<0.02) for all formulas, except formula no. 6 (O_cal__6_ = 285.3, p = 0.2217). Linear regression analysis showed good correlation between O_cal__1-8_ and O_meas_ (r range 0.6350-0.7173, p<0.0001, for all formulas). Bland-Altman analysis showed negative bias between O_cal_ and O_meas_, for all tested formulas, except for formula no. 3 (positive bias), the 95% confidence interval (95% Cl) was similar, regardless the formula used for osmolality calculation (Figure 1). The mean values of difference between O_cal_ obtained by formula no. 6 and no. 7 and O_meas_ were the lowest (-0.3 and -0.6 mOsm/kg H_2_O, respectively), whereas the mean value of difference between O_cal_ obtained by formula no. 8 and O_meas_ was the highest (-15.1 mOsm/kg H_2_O) in comparison with other formulas used in the study. According to Passing-Bablok regression, results obtained by formula no. 1 and no. 7 showed neither systematic nor proportional error compared to the measured value Table 4.

**Table 3:** The median values (interquartile range) of measured osmolality and calculated values obtained by the formulas in all samples and according to the age groups: babies from the first day to 3 months of life (group 1), babies between 3 months to 1 year (group 2), children between 1 and 2 years of life (group 3). The calculated values without statistically significant difference compared to the measured values are shaded. ^*^p = 0.2217, ^**^p = 0.3911, ^†^p = 0.0680, ^‡^p = 0.0717, compared to the measured value in each group<br>O_meas_ – measured osmolality, O_cal 1-8_ – osmolality calculated by formulas no. 1 to no. 8

	All<br>samples	Group 1	Group 2	Group 3
	Median [mOsm/kg H_2_O]<br>(Interquartile range)			
O_meas_	286.0<br>(283.0–289.0)	285.0<br>(282.0–290.0)	285.0<br>(282.0–288.0)	287.0<br>(282.0–289.0)
O_cal 1_	280.3<br>(277.9–283.6)	278.8<br>(275.2–284.0)	280.1<br>(277.6–282.6)	282.4<br>(279.8–285.4)
O_cal 2_	276.9<br>(274.7–280.0)	276.1<br>(273.0–279.4)	276.8<br>(274.7–279.6)	278.4<br>(276.0–280.8)
O_cal 3_	290.4<br>(287.6–293.4)	289.4<br>(285.0–294.5)	290.2<br>(287.5–291.9)	292.5<br>(289.5–294.8)
O_cal 4_	280.6<br>(278.0–283.3)	279.6<br>(275.7–284.4)	280.5<br>(277.9–282.1)	282.6<br>(279.8–284.9)
O_cal 5_	280.2<br>(277.8–283.0)	279.0<br>(275.3–283.9)	280.1<br>(277.4–281.9)	282.4<br>(279.6–284.8)
O_cal 6_	285.3^*^<br>(282.7–288.0)	284.3<br>(280.4–289.0)	285.2^**^<br>(282.6–286.9)	**287.4^†^<br>(284.5–289.6)**
O_cal 7_	284.8<br>(282.2–288.2)	283.2<br>(279.5–288.5)	**284.6^‡^<br>(282.1–287.1)**	286.9<br>(284.2–290.1)
O_cal 8_	270.3<br>(267.9–273.4)	268.9<br>(265.4–273.6)	270.1<br>(267.8–272.4)	272.2<br>(269.8-275.2)

**Table 4:** Regression equations according to Passing-Bablok comparing the measured osmolality with calculated values obtainedby different formulas. The fields with no systematic and proportional error for the results are shaded.

Formula<br>No.	All samples			Group 1			Group 2			Group 3		
	Regression<br>equation	Significant<br>difference		Regression<br>equation	Significant<br>difference		Regression<br>equation	Significant<br>difference		Regression<br>equation	Significant<br>difference	
		Intercept	Slope		Intercept	Slope		Intercept	Slope		Intercept	Slope
1	**y = 35.50 + 0.90 x**	**No**	**No**	y = 64.48 + 0.75 x	Yes	Yes	**y = -5.40 + 1.00 x**	**No**	**No**	**y = 38.03 + 0.85 x **	**No**	**No**
2	y = 48.80 + 0.80 x	Yes	Yes	y = 97.53 + 0.63 x	Yes	Yes	**y = -3.18 + 0.98 x**	**No**	**No**	y = 57.61 + 0.77 x	Yes	Yes
3	y = 32.37 + 0.90 x	Yes	Yes	y = 69.10 + 0.77 x	Yes	Yes	**y = 12.20 + 0.97 x**	**No**	**No**	**y = 31.39 + 0.91 x **	**No**	**No**
4	y = 39.37 + 0.85 x	Yes	Yes	y = 74.23 + 0.72 x	Yes	Yes	**y = 24.28 + 0.90 x **	**No**	**No**	y = 37.56 + 0.85 x	Yes	Yes
5	y = 33.84 + 0.86 x	Yes	Yes	y = 73.35 + 0.72 x	Yes	Yes	**y = 15.26 + 0.93 x**	**No**	**No**	y = 35.58 + 0.86 x	Yes	Yes
6	y = 42.10 + 0.85 x	Yes	Yes	y = 80.29 + 0.72 x	Yes	Yes	**y = 26.29 + 0.91 x**	**No**	**No**	**y = 38.80 + 0.87 x**	**No**	**No**
7	**y = 18.36 + 0.93 x**	**No**	**No**	y = 60.37 + 0.78 x	Yes	Yes	**y = -4.89 + 1.10 x**	**No**	**No**	**y = 33.66 + 0.89 x**	**No**	**No**
8	y = 28.79 + 0.85 x	Yes	Yes	y = 66.53 + 0.71 x	Yes	Yes	**y = 10.34 + 0.91 x**	**No**	**No**	y = 42.09 + 0.81 x	Yes	Yes

**Figure 2:**
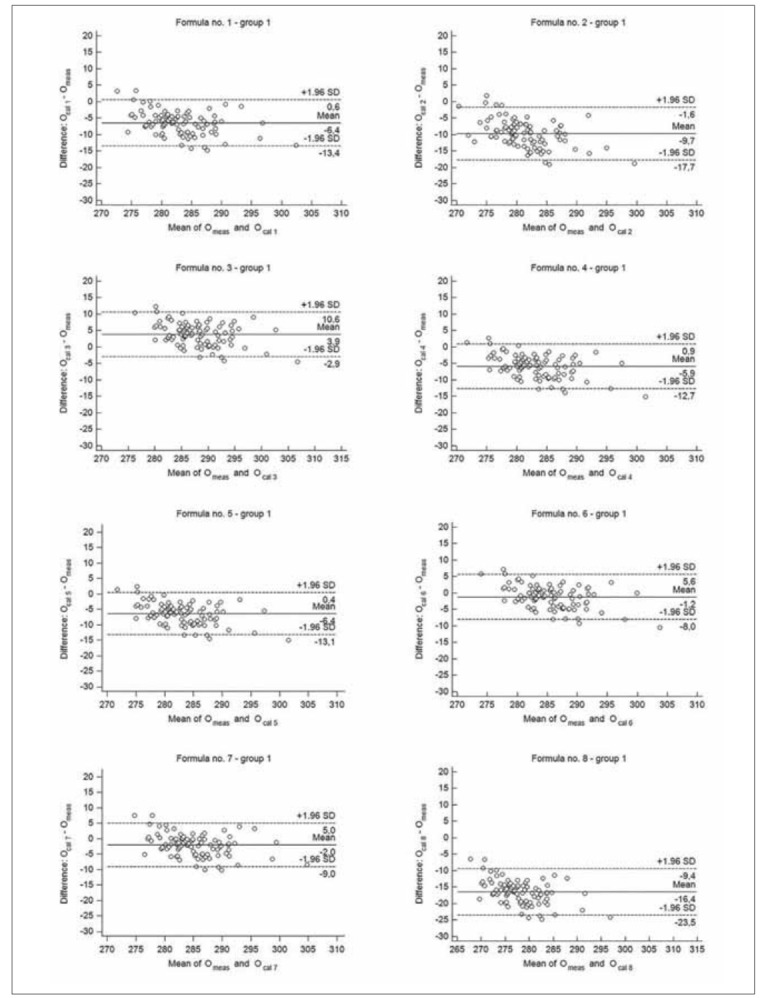
The Bland-Altman plots for the difference between measured osmolality (O_meas_) and calculated by different formulas (O_cal 1-8_) – group 1 (babies from the first day to 3 months of life).

**Figure 3:**
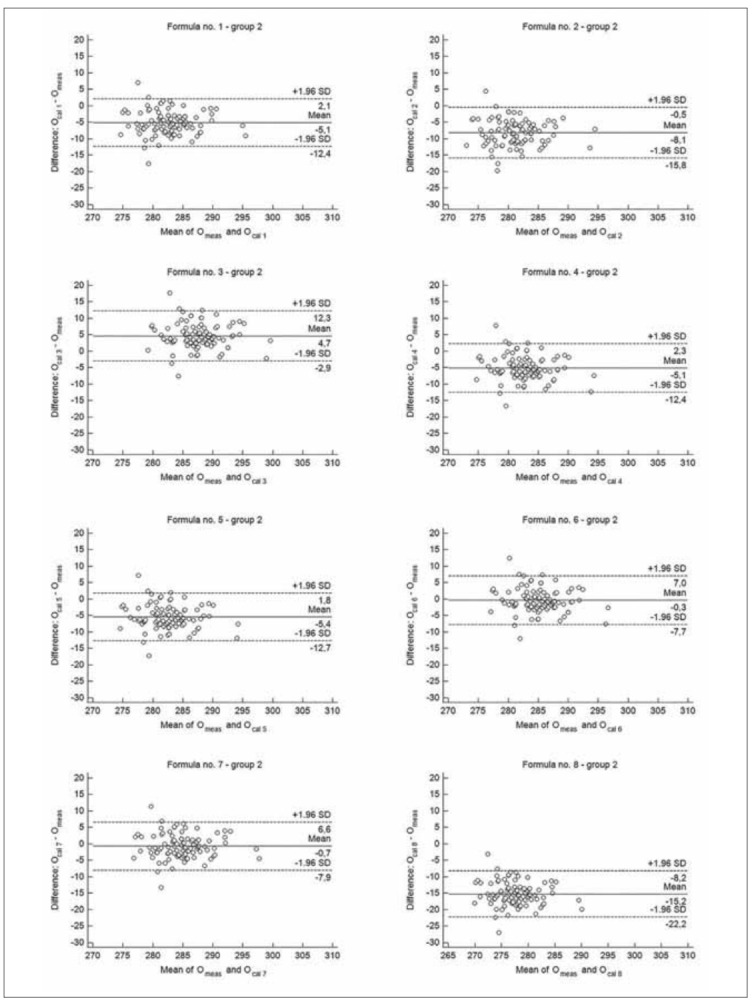
The Bland-Altman plots for the difference between measured osmolality (O_meas_) and calculated by different formulas (O_cal 1-8_) – group 2 (babies between 3 months to 1 year of life).

**Figure 4:**
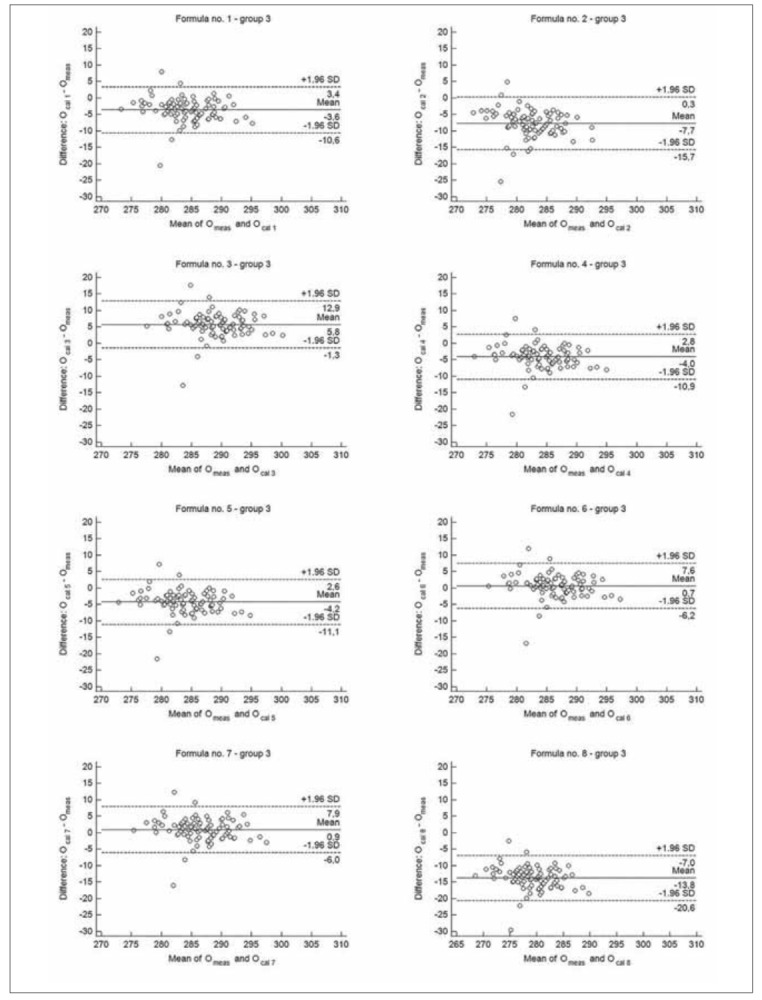
The Bland-Altman plots for the difference between measured osmolality (O_meas_) and calculated by different formulas (O_cal 1-8_) – group 3 (children between 1 and 2 years of life).

When results were compared according to the age groups, there were no significant difference between median values of measured osmolality and O_cal 6_ in group 2 and 3, as well as between median value of O_meas_ and O_cal 7_ in group 2 (Table 4). The median values obtained by each formulas showed good correlation with measured values. The correlation coefficients between O_cal 1-8_ and O_meas_ were from 0.7385 to 0.8241 in group 1 (p<0.00001, for all formulas), from 0.5900 to 0.6417 in group 2 (p<0.001, for all formulas) and from 0.5921 to 0.7121 in group 3 (p<0.02, for all formulas). According to Bland-Altman analysis, formula no. 6 and no. 7 showed the best performance for estimating osmolarity regardless the group. Mean difference between O_cal 6_ and O_meas_ ranged from -1.2 to 0.7 mOsm/kg H_2_O, whereas mean difference between O_cal 7_ and O_meas_ ranged from -2.0 to 0.9 mOsm/kg H_2_O in age groups (Figure 2, Figure 3 and Figure 4). The Bland-Altman analysis revealed also the highest bias for formula no. 8, regardless the analysed group. The total allowable error for the mean measured value of osmolality was 4.3 mOsm/kg H_2_O in each group. The difference between calculated and measured osmolality did not exceed this error only for values obtained by formula no. 6 and no. 7, in each analysed group, as well as when all samples were analysed (Figure 1, Figure 2, Figure 3 and Figure 4). In group 1 Passing-Bablok regression indicated both systematic and proportional error for results obtained by each formula compared to the measured values. In group 2 there were none of these errors for results obtained by each analysed formula, whereas in group 3 there were none of these errors only for results obtained by formula no. 1, no. 3, no. 6 and no. 7 (Table 4).

## Discussion

Osmolality is frequently used in describing water-electrolyte balance in the body and may help to identify the electrolyte disorders, which can be caused by many different conditions, such as kidney or heart diseases and some kinds of poisoning [9]. For example an imbalance of salt and water contribute to hypertension and chronic kidney disease. Hypernatremia and hyperosmolality is associated with high systolic blood pressure and decreased estimated glomerular filtration rate [15]. Osmolality depends on the number of osmotically active particles dissolved per kilogram of pure water (mOsm/kg H_2_O) [16]. The movement of water between the intracellular and extracellular water space is determined by osmolytes, mainly sodium ions and glucose. Increase of plasma glucose concentration generates an osmotic gradient which shifts water from the intracellular to the externalcellural volume, resulting in dilution extracellular solute, therefore also the sodium concentration. Serum osmolality can be measured directly, but due to the fact that osmometers are not available in all clinical laboratories, in daily medical practice, the calculated osmolality is more commonly used. Concentration of osmotically active analytes used for calculation of serum osmolarity are expressed in mmol/L, thus in fact osmolarity instead of osmolality is obtained [17]. In extremely diluted solutions, such as body fluids with slight fluctuations in their temperature and volume, it can be assumed that osmolarity and osmolality are roughly equivalent [18], thus in the present study we compare measured osmolality and calculated osmolarity without any converting factor. In published literature terms: osmolality and osmolarity are often used interchangeably or term osmolality describes both measured and calculated values.

Many formulas for serum osmolarity estimation have been previously described [12] [19] [20] and new ones are continuously proposed, but which of them reflect measured serum osmolality value is still unclear. Serum osmolality mainly depends on the sodium ion concentration as well as small osmotically active molecules such as glucose and urea, therefore these analytes are used in most formulas [21]. It should be remembered that urea can cross cell membrane and cannot be included in effective serum osmolality calculation. Different factors, e.g. 2; 1.89; 1.86, are used in different formulas to compensate the fact, that sodium solution is not completely dissociated in aqueous medium, moreover minor particles also contribute to osmolality and plasma contains only 93% water. Most formulas for osmolarity calculation were developed and validated for the adult population [22] [23] [24]. It is still too few proofs that in newborns and small children measured osmolality may be replaced by calculated value and it is still unknown which formula might be used.

In our study measured osmolality almost always resulted in greater values than the osmolarity calculated by equations, regardless analysed group. Only the median value of osmolarity calculated by formula no. 6: 1.86^*^(Na+K)+1.15^*^Glu+Urea+14 did not differ significantly from the measured osmolality value, in each analysed group of children excluding newborns up to 3 month of life. Furthermore, the median values obtained from all formulas showed good correlation with measured values. Bland-Altman plots showed quite similar 95% confidence interval regardless the formula used for osmolarity calculation however, the mean differences between the O_cal_ and O_meas_ values ranging from -16.4 to 5.8. The results of the published studies [25] [26] also showed, that calculated osmolarity values have good correlation with measured values. However, Kar et al. [27] demonstrated that the calculated osmolarity in pediatric population are statistically significantly higher than the measured values. There was no significant difference between measured osmolality and osmolarity calculated by formula: 2^*^Na+Glu+Urea (in our study formula no.1) and there was a significant positive correlation between analysed values.

The difference between the measured osmolality and calculated value is defined as osmol gap (OG - osmolality/osmolar gap) [17] and the value 10 mOsm/kg H_2_O was proposed as its reference limit [26]. OG is useful in detection of unmeasured exogenous serum osmotically active particles such as ethanol, methanol, ethylene glycol, mannitol or drugs. Different equations may give different osmol gaps, ranging even from -5 to 15 mOsm/kg H_2_O [18].

Over the years, it was demonstrated, that results obtained by one of the most widely used formulas: 2^*^Na+Glu+Urea, do not agree with true osmolality values in adult population [25], whereas might be a good equation for prediction of osmolality in pediatric population [28]. Some authors suggested, that this equation underestimates the calculated osmolarity and requires correction factors, to take into account other osmotically active substances found in the serum. But often these conclusions were made based on comparison the mean values of calculated and measured osmolality and correlation coefficients between calculated and measured values [25] [27]. The correlation coefficient describes how well the results of the two methods change together, therefore correlation describes only the relationship between two variables, not their compatibility and did not disclose systematic and proportional error between methods. According to the statistics recommendation r should not be used as an indicator of method acceptability. Linear regression analysis of our data showed for all tested formulas r less than 0.7173, so we used the paired data calculation, Bland-Altman analysis and Passing-Bablok regression.

Passing-Bablok regression, is not sensitive to outliers or the distribution of errors and allows to assess the systematic (intercept) and proportional (slope) differences between the two methods. When the 95% confidence interval for the intercept A contains the value 0, and 95% CI for the slope B contains the value 1, the conclusion is that one method yields sufficiently similar results to the second method [29]. In our study, the results obtained by of any of analysed formulas are burdened with systematic and proportional errors in group 1. On the contrary, in group 2 the calculated results were comparable to measured ones, for all analysed formulas. In group 3, only for formulas no. 1, no. 3, no. 6 and no. 7, there were no systematic or proportional errors between measured and calculated values. Ebonwu et al. [30] also used Passing-Bablok regression to evaluate the difference between measured osmolality and calculated values. They showed the proportionally negative bias for value obtained by most analysed formulas, including formula 2^*^Na+Glu+Urea (formula no. 1) and that this equation may need bias adjustment in individual population [30].

Results of our study are in agreement with the data of Munk et al. [23] as well as of Matrin-Calderon et al. [20] According to them, results obtained by the equation: 1.86^*^(Na+K) +1.15^*^Glu+Urea+14 (formula no. 6) were the most accurate, while the equation: 1.86^*^Na+Glu+Urea+9 (formula no. 8) should not be used in osmolality calculation. Similarly, Rasouli et al. [31] proved that the use of this formula 1.86^*^Na+Glu+Urea+9 (formula no. 8) tends to underestimate the true serum osmolality value, by about 10 ± 1 mOsm/kg H_2_O. The Bland-Altman analysis of our results also indicated the highest negative bias for osmolality calculated by formula no. 8 in each analysed group of results.

The present study is one of the very few studies comparing measured osmolality and calculated osmolality in newborns and small children. It is extremely difficult to choose the one formula for estimating serum osmolality which could be used for all children. The results of the published studies also do not clearly indicate which formula gives the most accurate results. Usually, the conclusions are strongly dependent on the statistical methods using in the study and their interpretation, as well as on both age and health status of the examined population [24] [26] [28]. Additionally, sodium shows dynamics changes during all periods of childhood [32]. Because the reference intervals for sodium are wide, especially in early infancy [33], it may cause that formulas do not give the accurate results.

Our results were also interpreted by analyses of acceptable differences between measured and calculated results using the total allowable error (TEa). According to quality criteria of the result, TEa for serum osmolality, derived from biologic variation, should not exceed 1.5% [14]. Therefore, the mean difference between calculated and measured osmolality was analysed. Only for values obtained by formula no. 6 and no. 7 these differences did not exceed 4,3 mOsm/kg H_2_O (that is 1.5% of the mean values), regardless the analysed group. Based on the requirements for total error for serum osmolality, it could be concluded that osmolality calculated by the following formulas: 1.86^*^(Na+K) +1.15^*^Glu+Urea +14 (formula no. 6) and 1.09^*^(1.86^*^Na+Glu+ Urea) (formula no. 7) provides to the same clinical decision as measured osmolality.

The limitation of the study might be the small number of the formulas used to calculate osmolality. We used the most common formulas selected from literature, easy to apply in clinical practise. The usefulness of the selected formulas should be also confirmed in external data set.

## Conclusions

To calculate osmolality in children aged between 3 months and 2 years old the following equation: 1.86^*^(Na+K) +1.15^*^Glu+Urea+14 might be used. Serum osmolality in children up to 3 month of life should be measured.

The present study is one of the few studies comparing measured serum osmolality and calculated osmolality in paediatric population. Many formulas for osmolality calculation have been already published, but there is still not clear recommendation which of them should be used in small babies. The message from the present study is:

-in children up to 3 month of life serum osmolality should be measured

-in children aged between 3 months and 2 years old, serum osmolality might be calculated by the following equation: 1.86^*^(Na+K)+1.15^*^Glu+Urea+14

## Dodatak

### Conflict of interest statement

All the authors declare that they have no conflict of interest in this work.

### List of abbreviations

O_meas_, measured osmolality;<br>O_cal_, calculated osmolarity;<br>HI, hemolysis index;<br>OG, osmol gap (osmolality/osmolar gap);<br>TEa, total allowable error.
